# Complementary Medicine Use and Perceptions of It in Victoria, Australia: A Statewide Cross-Sectional Survey

**DOI:** 10.3390/nu18071077

**Published:** 2026-03-27

**Authors:** Kaveh Naseri, Wejdan Shahin, Ayman Allahham, Hajira Bilal, Barbora de Courten, Thilini R. Thrimawithana

**Affiliations:** School of Health and Biomedical Sciences, RMIT University, Melbourne, VIC 3000, Australia; s3961858@student.rmit.edu.au (K.N.); wejdan.shahin@rmit.edu.au (W.S.); ayman.allahham@rmit.edu.au (A.A.); hajira.bilal@rmit.edu.au (H.B.)

**Keywords:** complementary medicines, supplements, consumer perceptions, safety, pharmacovigilance, health literacy

## Abstract

Background/Objectives: Complementary medicines (CMs) are widely used in Australia, yet consumer beliefs about their safety and effectiveness often diverge from the scientific evidence. Contemporary statewide data from Victoria, particularly about these perceptions and underlying perception profiles, are limited. We therefore aimed to characterise CM use patterns and perceptions of it among Victorian adults and identify the demographic and use-related belief patterns. Methods: A cross-sectional survey was conducted in metropolitan and regional Victoria (November 2024–August 2025) among adults (≥18 years) who had used complementary medicines in the previous 12 months (N = 447). The questionnaire assessed CM use patterns, perceived effectiveness, safety, quality, perceived risk relative to prescription medicines, adverse events, and demographics. The analyses included descriptive statistics, χ^2^ tests with multiple-comparison control, Spearman correlations, and a multivariable regression. An exploratory factor analysis (EFA) and latent class analysis (LCA) were used to identify the perception dimensions and distinct consumer profiles. Results: CM use was frequent (62.2% daily; 19.2% weekly) and often long term (>1 year, 55.0%). The most commonly used products were vitamin D (53.0%), multivitamins (39.8%), magnesium (34.5%), iron (33.8%), and vitamin C (30.0%). The perceptions were favourable: 77.3% rated CMs as effective, 90.4% as safe, and 60.3% as high quality; 78.5% perceived CMs to have lower side-effect risks than prescription medicines. Adverse events were reported by 12.3%. In the adjusted models, adults ≥ 65 years and monthly/occasional users were less likely to endorse “lower risk than prescription medicines” (aOR: 0.18; 95% CI: 0.06–0.51; aOR: 0.36, 0.18–0.72). East Asian respondents had lower odds of endorsing CM effectiveness than Caucasian/White respondents (aOR: 0.28, 0.11–0.72). Their perceived quality was higher among men (aOR: 1.73, 1.09–2.74) and adults aged 55–65 years (aOR: 3.81, 1.39–10.48). Conclusions: In this contemporary statewide Victorian sample, CM use was common and generally viewed positively, yet the comparative risk may be underestimated. Profiling perception patterns and identifying belief patterns by age, culture, and use intensity provides actionable targets for clinician/pharmacist counselling and culturally tailored education to support safer, evidence-aligned CM use.

## 1. Introduction

Complementary medicines (CMs), including vitamins, minerals, probiotics, prebiotics, herbal products, and nutritional supplements, are widely used and firmly embedded in contemporary self-care in Australia and worldwide [1,2,3]. The international literature similarly suggests that CM use is commonly shaped by its perceived benefits, perceived safety, and dissatisfaction with aspects of conventional care, although these drivers vary across settings and product types [3,4]. National surveys have consistently reported that around half of Australian adults use CM products, often alongside prescription medicines [5,6]. This broad product-based CM landscape includes nutrient supplements that are often self-selected, combined, and used long term, which may increase the risk of overlapping ingredients, unintended excess intake, and clinically relevant interactions with prescription medicines [7,8]. This raises important clinical and public-health concerns, as consumer beliefs about safety, efficacy, and quality do not always align with the available evidence, particularly when commonly used supplements are perceived as routine or inherently low risk. Despite their widespread use, evidence of benefit varies across products and indications, adverse effects and clinically meaningful drug–CM interactions may be under-recognised, and contamination or variable quality continues to be reported [9,10,11]. These issues are amplified by inconsistent international regulatory standards and the frequent nondisclosure of CM use to health professionals [5]. They are further amplified by limited understanding of herb/supplement–drug interactions and by the fact that cumulative exposure can occur when the same nutrient is present across multiple products [7,12]. Recent Australian regulatory action on vitamin B6 further illustrates the policy relevance of this issue, as reports of peripheral neuropathy associated with high-dose and cumulative supplement exposure have led to stronger safety controls for higher-dose products [13].

Despite these concerns, CM use remains common. The contributing factors include perceptions of lower risk than for prescription medicines [14]. These beliefs are often tied to the assumption that “natural” products are inherently safer and less likely to cause side effects [3]. Other drivers include dissatisfaction with the effectiveness or side effects of prescription medicines [15] and social influences, such as recommendations from family/friends and widespread social media exposure [3,15,16]. The international systematic review evidence also suggests that the expected benefits and perceived safety are among the most commonly reported reasons for CM use, alongside dissatisfaction with conventional care and social influences [3]. Together, these drivers encourage CM use as adjuncts to, or substitutes for, conventional care. In some contexts, these beliefs may also contribute to delayed professional assessment or delayed use of conventional care, particularly when symptoms are self-managed, interpreted as minor, or attributed to causes perceived as manageable without medical review [17,18]. The Australian Poisons Information Centre data, hospital-based liver injury case series and regulatory safety alerts have documented hepatotoxicity, heavy metal poisoning and other harms associated with some products [19,20].

In Australia, the Therapeutic Goods Administration (TGA) regulates CM products via a risk-based, two-tier framework. There is no separate Victorian registration system for CMs. Products supplied in Victoria are regulated nationally through the TGA and the Australian Register of Therapeutic Goods (ARTG), while Victoria mainly oversees medicines and poisons controls, such as their possession, storage, prescribing, and supply. Most CM products are *listed* (AUST L) rather than *registered* (AUST R) [21]. *Listed* medicines may contain only permitted low-risk ingredients from the TGA permissible ingredients list, may make only low-level indications, and must be manufactured in accordance with Good Manufacturing Practice, which sets baseline quality and safety standards [22]. Importantly, “lower risk” in this regulatory context does not mean risk-free, because listed products may still cause adverse effects, interact with conventional medicines, or be used inappropriately by consumers. In addition, product-related risks may arise from contamination, adulteration, substitution, batch-to-batch variability, and inconsistent quality control or labelling, all of which may affect both safety and consumer confidence in product quality [23]. In addition, the labels for listed medicines are not individually pre-approved before marketing, and current requirements do not routinely mandate drug–CM interaction warnings or consumer medicines information. Consequently, when co-use with prescription medicines is common and disclosure to clinicians is inconsistent, consumer perceptions and decision-making become key determinants of risk [22].

In this context, understanding consumer beliefs is central to safer CM use. Their perceptions about CM effectiveness, safety, quality, and comparative risk versus prescription medicines shape whether people initiate, combine, or persist using products. They may also influence disclosure to healthcare professionals (HCPs), responsiveness to counselling, and where consumers seek information and make purchases. These beliefs, however, are not uniform and may vary by age, gender, cultural background, health status, and frequency of use [6]. Yet the contemporary state-level data needed to inform targeted action, particularly in Victoria, remain sparse. In addition, most available surveys summarise perceptions at the item level, offering limited insight into whether beliefs cluster into distinct, actionable perception profiles.

Victoria is one of Australia’s most culturally diverse and populous states, yet the contemporary state-level evidence on public perceptions of CMs remains limited. This constrains the ability to identify higher-risk groups, such as frequent users who do not consult with HCPs, individuals with strong beliefs about CMs’ superior safety or effectiveness, and people taking prescription medicines for chronic conditions who face elevated interaction risks. It also limits the development of tailored educational strategies, professional guidance, and policies that could effectively address local patterns of use and risk. Addressing this information gap is a prerequisite for pragmatic, culturally responsive strategies that align consumer behaviour with evidence and regulatory safeguards.

To address this gap, we conducted a statewide, cross-sectional survey of adults across metropolitan and regional areas. The study aimed to characterise patterns of CM use; assess perceptions of their effectiveness, safety, quality, and comparative side-effect risk versus prescription medicines; and identify the socio-demographic and use-intensity correlates. These findings may help inform clinician counselling, public health messaging, and regulatory communication. We also applied an EFA and LCA to derive the underlying perception dimensions and identify the distinct perception profiles among Victorian CM users, providing a contemporary baseline to support targeted public health and clinical messaging. In this study, “CMs” refer to product-based complementary medicines (vitamins, minerals, probiotics, prebiotics, herbal medicines, and nutritional supplements) and excludes practitioner-delivered therapies (e.g., acupuncture, chiropractic, massage) and broader integrative care.

## 2. Materials and Methods

### 2.1. Study Design and Setting

A statewide cross-sectional survey was conducted, using non-probability, venue-based purposive sampling across metropolitan and regional Victoria (19 November 2024–25 August 2025). Recruitment occurred both online (including quick response [QR] codes and social media posts) and in person at community settings, such as pharmacies, libraries, gyms, health clinics, and senior centres. Field visits were conducted across 21 cities and towns in Victoria to recruit participants directly and to ensure coverage of both metropolitan and regional areas. A probability sample of recent CM users was not feasible because no population sampling frame of Victorian adults who had used CMs within the previous 12 months was available. The study therefore prioritised broad geographic coverage and heterogeneity of current CM users rather than population-weighted prevalence estimation.

### 2.2. Participants

The eligible participants were adults (≥18 years) residing in Victoria, Australia, who reported CM use within the previous 12 months and could complete an English-language questionnaire. Before starting the survey, potential participants viewed an online or printed information statement outlining the study objectives, voluntary and anonymous participation, estimated completion time, and confidentiality safeguards. Proceeding to the survey signified informed consent as approved by the human research ethics committee. The exclusion criteria were an age under 18 years, no CM use in the past 12 months, residence outside Victoria, insufficient English proficiency, or declining to proceed. Eligibility was enforced via screening questions at the survey’s entry. At the end of the survey, respondents were invited to enter an optional draw for an AUD $20 voucher. To preserve anonymity, contact details for the draw were collected in a separate Qualtrics form that could not be linked to the survey responses and were used solely for prize notification.

### 2.3. Survey Instrument

A 41-item anonymous survey was developed in Qualtrics XM (Qualtrics, Provo, UT, USA), covering CM use patterns; purchase and information sources; perceptions of effectiveness, safety, and quality (including comparisons with prescription medicines, side-effect risk, and preferences for chronic/minor conditions); adverse reactions; label use; engagement with HCPs; and demographics/health. The response formats included multiple-choice items, five-point Likert-type scales, ranked items, and optional free text. The instrument was purpose-built for this study and pilot-tested (n = 15) for clarity, burden, and sensitivities; the wording and demographics were refined and a <15 min completion time was targeted. The questionnaire was not externally validated against dispensing, prescribing, or clinical outcome data. Accordingly, the perception items should be interpreted as self-reported beliefs rather than objective measures of factual accuracy. In this manuscript we analyse the demographics, usage patterns, and perception domains; the other domains are reported separately (full instrument: Supplementary File S1).

### 2.4. Data Management

The records were screened to remove incomplete entries and potential duplicates. The eligibility screeners (age ≥ 18 years, CM use in the past 12 months, Victorian residence) were enforced at the survey entry. Potential duplicate entries were reviewed using available survey metadata and response pattern checks; where two records were judged likely to represent the same response, the more complete entry was retained. The missingness was summarised before the analysis dataset was finalised. The data will be stored on secure RMIT servers with restricted access for seven years.

### 2.5. Variables

The primary perception outcomes were perceived effectiveness, perceived safety, perceived quality, and comparative side-effect risk of CMs relative to prescription medicines. The five-point scale perception items were collapsed into three categories: Disagree (1–2), Neutral/Unsure (3), and Agree (4–5). The comparative risk vs. prescription medicines was coded as Higher/About the Same/Lower. CM use frequency was classified as daily, weekly, monthly, or occasional (<once/month) and, when prespecified, was collapsed to daily/weekly versus monthly/occasional to reduce sparsity. Daily prescription medicine use was coded as Yes/No. Ethnicities were grouped (Caucasian/White/European; Middle Eastern/North African; South Asian; East Asian) to minimise sparse cells. The covariates considered in the multivariable analyses included age group, gender, education, ethnicity, CM use frequency, self-rated health, prescription medicine use, and presence of ≥1 ongoing medical condition. The analyses used available-case handling; the valid Ns are shown in the tables/figures. For the EFA and LCA, only the surveys of respondents with complete data on the included perception items were analysed.

### 2.6. Statistical Analysis

We used descriptive statistics for demographics, health status, CM use patterns, and perceptions. Categorical associations used Pearson’s χ^2^ with Cramér’s V. For significant tables, cellwise differences used adjusted standardised residuals with Holm correction. Where expected counts were <5, we confirmed results by collapsing categories and/or using Fisher’s exact test. Associations involving ordered variables used Spearman’s ρ.

An ordinal logistic regression modelled the perceived effectiveness, safety, and quality. The adverse reaction reporting used a binary logistic regression. For the side-effect risk vs. prescription medicines, sparse ‘Higher risk’ responses motivated a prespecified primary model collapsing the outcome to Lower vs. About the Same/Higher; we report the adjusted odds ratios (ORs, 95% CIs). Sensitivity analyses (excluding ‘Higher’ responses; multinomial model: Higher vs. Same; Lower vs. Same) were run where feasible. The proportional odds assumptions were assessed (score tests; partial residual checks) and were not meaningfully violated. The multicollinearity was low (variance inflation factors < 2.5). Multiple comparisons were controlled using a 5% false discovery rate (Benjamini–Hochberg); a Holm-adjusted ASR identified the contributory cells in significant tables.

We examined nine perception items using exploratory analyses. An EFA was conducted on complete cases for the included perception items using rank-transformed data and a Spearman-equivalent correlation matrix; factor retention was guided by a parallel analysis and scree inspection, and the factors were extracted by principal axis factoring with a Promax rotation. The salient loadings were defined as |loading| ≥ 0.30. Because these analyses were exploratory and based on reduced complete-case samples, the factor solutions were interpreted cautiously and used for descriptive profiling rather than as primary inferential outcomes. An LCA was conducted using the same perception items, recoded as Disagree/Neutral/Unsure/Agree. For the comparative risk item, the categories were Higher, About the Same, and Lower. Two- to five-class solutions were estimated using expectation–maximisation with multiple random starts and weak Dirichlet smoothing. The model selection prioritised BIC, entropy, and class interpretability, and solutions containing classes with <10% of the sample were not retained. The exploratory analyses compared HCP-recommended vs. non-HCP pathways and consultation rates among prescription medicine users, with BH–FDR control (Software: SPSS v29 and Python 3.9).

An a priori sample size calculation was used as a pragmatic planning guide for the main multivariable analyses. Using G*Power (Version 3.1.9.7) for a small-to-moderate effect (f^2^ = 0.05), α = 0.05, 8 predictors, and 90% power, the target sample size was 399. Given the non-probability design, all estimates are unweighted and should not be interpreted as population-weighted estimates for Victorian adults.

This study is reported in accordance with the Strengthening the Reporting of Observational Studies in Epidemiology (STROBE) statement for cross-sectional studies [24]; the completed checklist is provided in Supplementary File S2.

## 3. Results

### 3.1. Participant Characteristics

**Figure 1:**
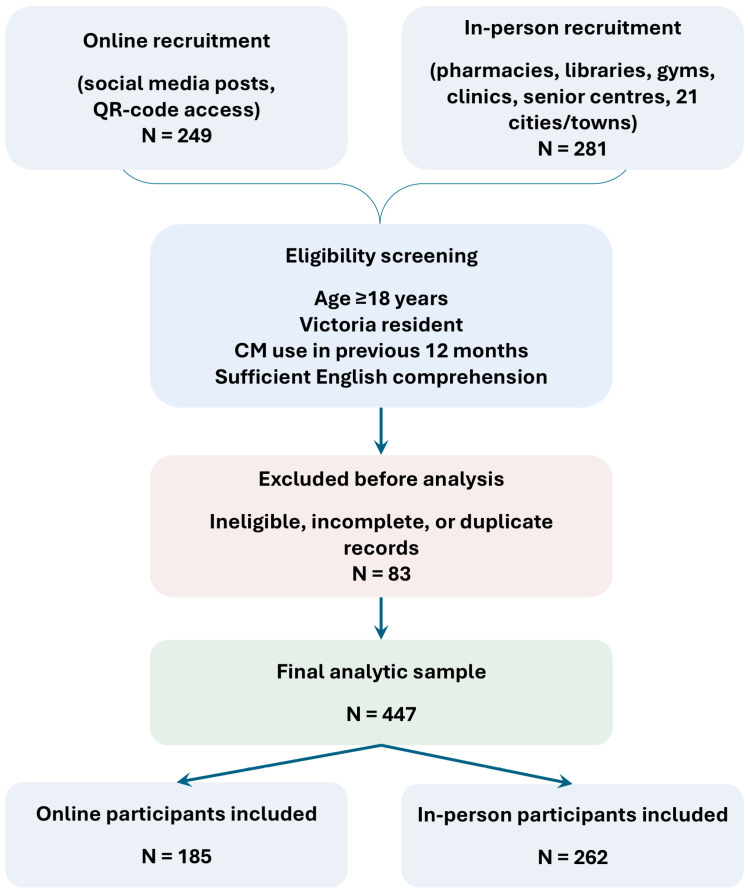
Participant recruitment flowchart for the complementary medicine survey in Victoria, Australia. Note: The approach, refusal, and survey-opening counts were not systematically recorded for the anonymous online dissemination and field-based recruitment; therefore, a participation denominator could not be established.

A total of 447 participants (262 in person; 185 online) completed the survey. A recruitment flowchart summarising the two recruitment streams and the final analytic sample is provided in Figure 1. Of the participants (valid n = 447), 64.0% were female, 33.8% were male, and 2.2% identified as another gender or preferred not to disclose their gender. Their ages were broadly distributed (valid n = 432), with the largest strata being 18–24 y (30.3%) and 35–44 y (22.0%). Most participants resided in metropolitan Victoria (319/447, 71.4%), while 128/447 (28.6%) lived in regional areas (closely reflecting the Victorian population distribution). Their ethnic backgrounds (valid n = 442) were diverse, most frequently Caucasian/White (38.0%), Middle Eastern/North African (21.7%), South Asian (16.5%), and East Asian (7.2%). Their education levels (valid n = 446) were high-school or less (26.9%), undergraduate (34.5%), and postgraduate (38.6%). Most participants rated their health status (valid n = 446) as excellent/good (74.2%); 50.1% reported at least one ongoing health condition, most commonly back pain (19.9%), mental health conditions (14.5%), and asthma/respiratory conditions (8.3%). Daily prescription medicine use was reported by 43.2% of the participants (see Table 1). Because the sample was non-probability and unweighted, these estimates should not be interpreted as population representative of Victorian adults.

### 3.2. Patterns of CM Use

CM use was frequent and sustained (Table 2). More than 80% of participants (valid n = 447) used CMs daily (62.2%) or weekly (19.2%), and 55.0% reported their use for more than one year. The most frequently used products were vitamin D (53.0%), multivitamins (39.8%), magnesium (34.5%), iron (33.8%) and vitamin C (30.0%); extended product lists are shown in Supplementary Table S1. CM use was commonly recommended by health care professionals (39.9%), but most products were initiated via a self or non-professional recommendation (60.1%).

### 3.3. Perceptions of CMs and Medicines

**Figure 2:**
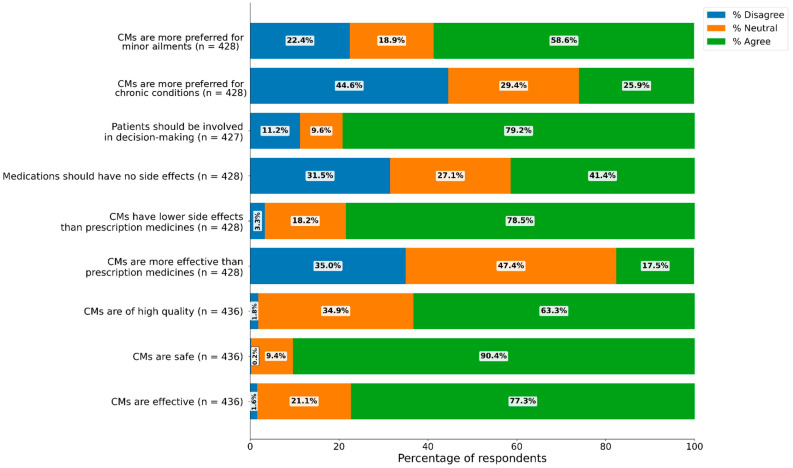
Consumer perceptions of CMs. Percentages are calculated within each item using valid responses only, and item-specific valid n values are shown in the figure labels. Percentage values for each response category are displayed within corresponding bar segments. Responses originally collected on five-point scales are collapsed into three categories: agreement (positive responses), neutrality (including “don’t know/unsure”), and disagreement (negative responses).

Most respondents (valid n = 436) rated CMs as effective (77.3%), safe (90.4%), and of high quality (60.3%). Nearly four in five perceived CMs as having a lower risk of side effects than prescription medicines (valid n = 428) (78.5%), whereas only 17.5% considered CMs more effective than prescription medicine (Figure 2).

Attitudes toward medication side effects were mixed. In response to the statement that “medications should have no side effects,” (valid n = 428) 41.2% agreed, 27.1% were neutral/unsure, and 31.6% disagreed (percentages may not sum to 100 due to rounding). Notwithstanding this division, the respondents converged on the importance of patient involvement in treatment decisions, including the use of CMs. The majority supported shared decision-making (valid n = 427) (78.6%), with 9.6% neutral and only 11.2% disagreeing. The preferences were severity-dependent (valid n = 428): 44.7% preferred prescription medicines and 26.0% CMs for chronic conditions, whereas 58.6% (valid n = 428) preferred CMs for minor ailments (see Figure 2).

### 3.4. Healthcare Professional (HCP) Involvement and Purchasing Patterns

The participants reporting an HCP recommendation for CM use (39.9%) did not differ from the others in their overall perceptions after a multiple-comparison adjustment (all *q* ≥ 0.05). However, they were more likely to purchase CMs at pharmacies (aOR: 3.68; 95% CI: 1.90–7.14) and reported a narrower product mix (lower odds of using multivitamins, collagen, zinc, and glucosamine; all *q* ≤ 0.05). Among the prescription medicine users (n = 193), around 70% reported consulting their physician before taking CMs. The consultation rate was modestly higher among women than men (73.7% vs. 58.8%; χ^2^ = 6.01; *p* = 0.049; Cramér’s V = 0.18) and showed no FDR-surviving differences across the other demographics. Overall, HCP involvement appeared to influence purchasing channels and product selection more than underlying beliefs.

### 3.5. CM-Related Adverse Effects

Among the respondents with valid data (n = 430), 53 (12.3%) reported experiencing a CM-related side effect, while 47 (10.9%) were unsure. Among those who reported adverse effects, most described them as mild (39/53, 73.6%), followed by moderate (8/53, 15.1%), and severe (6/53, 11.3%). In the adjusted models, reporting at least one medical condition was associated with higher odds of an adverse event (aOR: 2.90; 95% CI: 1.26–6.67; *p* = 0.012). Higher odds were also observed among probiotic users (aOR: 3.39; 95% CI: 1.31–8.81; *p* = 0.012) and iron users (aOR: 2.30; 95% CI: 1.00–5.28; *p* = 0.050). The participants aged 45–54 years had lower odds of reporting a side effect compared with younger adults (aOR: 0.16; 95% CI: 0.03–0.93; *p* = 0.041). Higher self-rated health (on a five-point scale from “terrible” to “excellent”) was positively associated with side effect reporting (aOR per category: 2.32; 95% CI: 1.34–4.01; *p* = 0.003), possibly reflecting increased awareness or help-seeking among people more engaged with their health.

### 3.6. Associations Between Demographics and Perceptions

**Figure 3:**
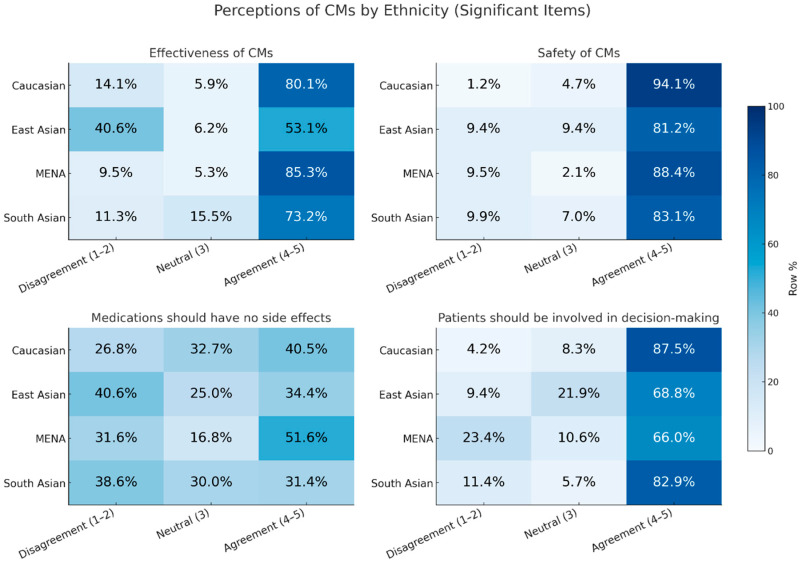
Ethnic differences among selected CM perceptions among Caucasian/White, Middle Eastern/North African (MENA), South Asian, and East Asian participants. Responses are collapsed into disagree, neutral/unsure, and agree. Values shown are row percentages based on valid respondents from each ethnic group; the colour scale (Row %) indicates the proportion selecting each response.

The bivariate associations between demographics, CM use frequency and the nine perception items are summarised in Table 3. The Spearman correlations between the four-level CM-use-frequency variable and the nine perception items are reported in Supplementary Table S2. After the FDR adjustment, age was associated with several perceptions, including overall effectiveness, comparative effectiveness versus prescription medicine, comparative side effect risk, and the belief that medications should have no side effects. Ethnicity was associated with perceived effectiveness and support for patient involvement in decision-making. Self-rated health was associated with perceived effectiveness and safety, and CM use frequency was associated with comparative side effect risk (all *q* < 0.05 within predictor families). The cellwise post-hoc analyses (Supplementary File S3) indicated that agreement that CMs are effective was lowest among participants aged 18–24 years and lower among East Asian respondents than among Caucasian/White respondents. The ethnic differences in selected CM perceptions are summarised in Figure 3. Because all the participants were CM users, the ethnicity analyses should be interpreted as differences in perceptions within the CM-using sample rather than as differences in population-level CM use prevalence. The respondents with fair or poor self-rated health were less likely to agree that CMs are effective or safe than those reporting better health. For the comparative side effect risk, frequent CM users were more likely to rate CMs as having lower side effect risks than prescription medicines, whereas less frequent users and adults aged 65 years or older were more likely to select “same or higher risk”. The belief that medications should have no side effects was more strongly endorsed among younger adults.

### 3.7. Multivariable Analyses

In the ordinal logistic regression models, older age groups had higher odds of endorsing greater CM effectiveness compared with adults aged 18–24 years, whereas less frequent CM users (monthly/occasional) had lower odds (Table 4). East Asian ethnicity and postgraduate education were also associated with lower odds of higher effectiveness ratings. For perceived safety, postgraduate education (vs. high school or less) was associated with lower odds of agreement that CMs are safe (aOR: 0.33; 95% CI: 0.11–0.97). For perceived quality, adults aged 55–65 years (vs. 18–24 years; aOR: 3.81; 95% CI: 1.39–10.48) and men (vs. women; aOR: 1.73; 95% CI: 1.09–2.74) reported higher perceived product quality. In the primary binary model of comparative side effect risk (lower vs. same/higher), less frequent CM users were less likely to endorse CMs as “lower risk” than more frequent users (aOR: 0.36; 95% CI: 0.18–0.72). Adults aged 65 years or older were also less likely to endorse this view (aOR: 0.18; 95% CI: 0.06–0.51). The other covariates were not significant. The sensitivity analyses using multinomial models (higher vs. same, lower vs. same) yielded directionally consistent inferences (Supplementary Table S3).

### 3.8. Exploratory Factor Analysis (EFA)

**Figure 4:**
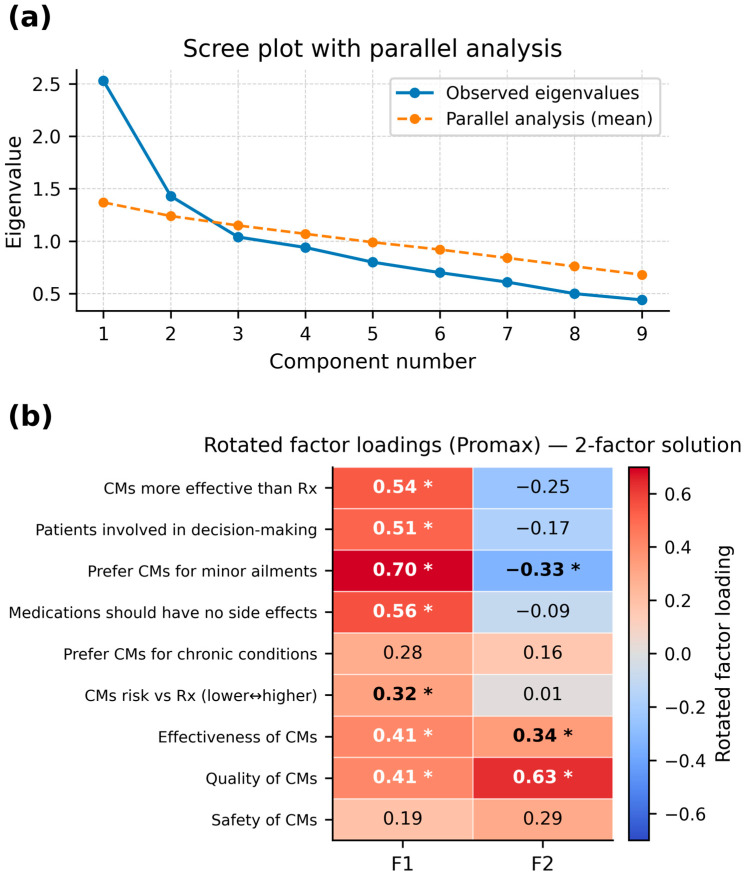
Exploratory factor analysis of nine CM perception items. (**a**) Scree plot with parallel analysis benchmark: The observed eigenvalues (solid line) from the Spearman-equivalent correlation matrix are plotted against the parallel analysis reference (dashed line). The first two observed eigenvalues exceed the benchmark, supporting a two-factor solution for the nine items in Table 3. (**b**) Promax-rotated factor-loadings heatmap: Each cell shows a rotated loading; each |loading| ≥ 0.30 is highlighted (bold with an asterisk). Factor 1 reflects CM preference/autonomy and comparative beliefs; Factor 2 reflects trust in CM quality and safety. Positive loadings indicate stronger endorsement of the construct. Total variance explained: 37.3% (Factor 1: 24.8%; Factor 2: 12.5%) (CM = complementary medicine).

An exploratory factor analysis of the complete cases was conducted for the nine perception items (N = 168), and identified a two-factor structure explaining 37.3% of the variance (KMO = 0.70; Bartlett’s *p* < 0.001). Factor 1 captured the preferences and comparative beliefs about CMs. It included greater perceived effectiveness, lower perceived side effects, preference for CMs for minor ailments, and autonomy in decision-making (α = 0.69). Factor 2 reflected trust in CM quality and safety. Because this factor comprised only two items and had low internal consistency, it should be interpreted descriptively and cautiously (α = 0.33). The scree and parallel analysis plots are shown in Figure 4a,b.

### 3.9. Latent Class Analysis (LCA)

**Figure 5:**
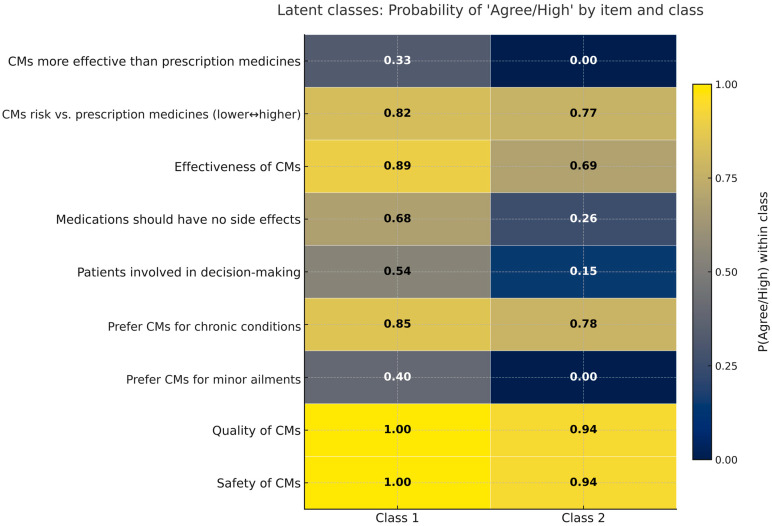
Latent classes of complementary medicine perception profiles (heatmap, cividis palette). Probability of “Agree/High” for each perception item within each latent class from the selected two-class model (cell values shown). Class proportions: 63.7% and 36.3%; entropy = 0.791; N = 178. The items include perceived effectiveness, safety, quality, comparative effectiveness (“CMs more effective than prescription medicines”), decision-making involvement, preference for CMs for chronic and minor conditions, the belief that medications should have no side effects, and comparative risk vs. prescription medicines. Full (disagree/neutral/agree) probabilities are reported in Supplementary Table S4.

A latent class analysis of the complete cases was conducted for the included perception items (N = 178). A two-class solution was selected (entropy = 0.791), distinguishing a larger “Enthusiast” group with stronger endorsement of CM effectiveness, safety, quality, and more frequent CM use, from a “Skeptical/Cautious” group with more moderate beliefs and less frequent use. However, both classes generally rated CM quality and safety positively and tended to view CMs as lower risk than prescription medicines. Given the reduced complete-case sample and the exploratory purpose of this analysis, these class profiles should be interpreted as hypothesis generating rather than definitive (Figure 5, Supplementary Table S4, Supplementary Figure S1).

## 4. Discussion

In this first statewide study of CM perceptions in Victoria, adult CM users generally held favourable views of these products, particularly regarding safety. To our knowledge, this study also adds a novel analytic perspective by using both an EFA and LCA to move beyond item-level summaries and identify interpretable perception dimensions and consumer profiles in a contemporary Victorian sample. Most respondents rated CMs as effective and safe, and many indicated they would choose CMs for minor ailments while preferring prescription medicines for chronic conditions. These patterns align with Australian surveys and international work showing that people often consider herbal and complementary products relatively low risk even when they do not view them as more effective than conventional medicines [5,6,25,26,27]. These findings are also broadly consistent with the wider international literature showing that CM use is often shaped by perceptions of benefit, naturalness, and relative safety, although the strength and expression of these beliefs vary across populations and product types [3,4,28]. At the same time, limited understanding of herb or supplement–drug interactions and inconsistent disclosure to health professionals remain important ongoing safety concerns [3,12]. In addition, CM-related adverse effects and subgroup differences in comparative risk beliefs underline the need for clearer, product-specific risk communication and routine clinical enquiry about CM use.

Although this study did not directly measure delays in diagnosis or healthcare seeking, strong beliefs about CM effectiveness and their low comparative risk may, for some consumers, encourage prolonged self-management before seeking professional assessment, particularly if symptoms are persistent but initially perceived as minor. This possibility reinforces the importance of counselling that distinguishes appropriate self-care from situations in which timely medical review is warranted [17,18]. This is particularly relevant for commonly self-selected nutrient products, because the overlapping use of multivitamins, single vitamins/minerals, and other wellness supplements may increase the risk of excess intake or drug–supplement interactions, even when the products are perceived as routine or low risk [7,12].

Relatedly, work published in Patient Preference and Adherence [29] suggests that these perceptions are not isolated beliefs but part of a broader attitudinal orientation toward CMs. In that Malaysian population survey, stronger CM-related beliefs were correlated with more positive attitudes toward CM use, and CM users showed stronger belief/attitude profiles than non-users. This matters clinically because stronger pro-CM orientations can coexist with low perceived risk and may reduce motivation to disclose their use to health professionals, which can increase the likelihood of avoidable interactions and harms.

Taken together, two patterns are particularly relevant for practice. First, the comparative risk beliefs diverged from the comparative effectiveness judgements. Many participants viewed CMs as lower risk than prescriptions, but far fewer judged them as more effective. This asymmetry may help explain the observed preference split for their substitution for minor problems but persistence with prescribed therapy for chronic disease. Public health communication that acknowledges their perceived safety while correcting the under-recognition of specific risks (e.g., product–drug interactions, product-specific adverse effects) may be more credible, and thus more effective, than messaging that dismisses their perceived benefits outright.

Second, the gradients by age and use intensity suggest different psychological models of risk. In the adjusted analyses, adults aged 65 years or older and less frequent CM users were substantially less likely to endorse the belief that CMs carry lower side effect risks than prescription medicines. In contrast, frequent users tended to rate CMs more favourably. This pattern is consistent with cognitive consistency and confirmation processes [30], whereby people tend to keep beliefs aligned with past choices and to preferentially seek/recall or interpret information that supports existing views. One possible mechanism for the attenuated “lower risk” belief among older adults is exposure: older people more often take prescription medicines, and their concomitant use increases exposure to counselling about interactions and adverse effects, tempering the “natural = safer” assumption, even when the perceived CM effectiveness remains high. Clinically, this matters because concurrent use is common and most consequential in older adults [5,6].

The estimates of this study fit within the Australian literature. National surveys consistently show high CM product use (around half of adults); frequent same-day co-use with prescription medicines (especially among older people); and predominantly positive, sometimes optimistic, perceptions of safety [6,9]. The Victorian pattern of endorsement for CM use for minor ailments, but not chronic conditions, echoes national findings that self-care motivations, perceived gentleness, and desire for autonomy drive CM use, while trust in prescription medicines persists for long-term disease management [6,9]. The present study extends that picture to the state level by highlighting the heterogeneity by age, ethnocultural grouping, and use intensity. It also shows how multiple perception domains may cluster into actionable belief patterns through an EFA and LCA. The lower adjusted odds of East Asian respondents endorsing CM effectiveness may reflect differences in product familiarity, preferred information channels and languages, or varying expectations of what counts as “effective”. They may also reflect differences in the mix of products used across groups. Relatedly, the respondents who reported HCP-advised use tended to purchase via pharmacies and reported a narrower product mix, despite similar overall frequency and duration of CM use.

As exploratory extensions of the main survey analyses, the EFA and LCA suggest that CM perceptions may cluster into a small number of broader dimensions and user profiles. Because these analyses are based on reduced complete-case samples, they should be interpreted cautiously and viewed as hypothesis generating. Nonetheless, the pattern is internally coherent: one dimension reflects preference and comparative beliefs, while another reflects trust in CM quality and safety, and the LCA distinguishes a more enthusiastic profile from a more cautious one. These preliminary profiles may be useful for designing more targeted education and public health communications, because audience segmentation can help align messages with differing beliefs, risk perceptions, and information needs. In that sense, the EFA/LCA findings may offer a pragmatic starting point for evidence-informed communication strategies and policy design. However, they require confirmation using larger datasets with more complete item responses before they can be used as a basis for formal intervention targeting or policy segmentation [31].

Approximately 12% reported adverse effects. The inclusion of severity grading adds important context to this finding, as it helps distinguish minor self-limiting effects from events of greater potential clinical significance. At the same time, the observed proportion is still likely to underestimate the true burden of CM-related harm. Underreporting may occur because consumers do not always recognise a symptom as related to a CM, may interpret mild or expected effects as too minor to mention, may stop the product without seeking advice, or may be unaware of formal adverse-event reporting pathways. Attribution may be particularly difficult when CMs are used alongside prescription medicines or with the presence of chronic conditions. The exploratory models suggest higher reporting among respondents with any chronic condition and among probiotic and iron users, patterns that are clinically plausible. Oral iron frequently causes gastrointestinal (GI) adverse effects including constipation, dyspepsia, and abdominal pain. Meta-analyses have shown increased GI side effect odds versus placebo or IV iron, although the dose form and regimen adjustments may improve tolerability [32,33,34,35]. In practice, counselling may help by anticipating and normalising gastrointestinal symptoms, and, where clinically appropriate, discussing strategies that could improve tolerability (e.g., adjusting dose, formulation, or dosing frequency) and providing clear thresholds for stopping a product and seeking review. For probiotics, randomised trials for common indications (e.g., IBS) generally report low rates of serious adverse events, although mild GI symptoms can occur; strain-specific effects and vulnerable host factors (e.g., severe immunosuppression) should be considered when advising use [32,36,37].

From a regulatory and consumer protection perspective, our findings may partly reflect the influence of Australia’s current risk-based framework for CMs, in which most products are listed rather than registered medicines and are not individually evaluated for efficacy before market entry; however, this regulatory classification does not mean such products are risk-free for consumers [6,21,22,38]. This also aligns with broader international discussions around traditional and complementary medicine products, in which issues of standardisation, safety evaluation, manufacturing consistency, and regulatory oversight remain important for public confidence in product quality and safety [39]. Optimistic comparative risk beliefs, alongside observed adverse effects, suggest a need for clearer label-based and consumer-facing information about product-specific risks, particularly for groups at higher risk of interactions or adverse events. Recent Australian action on vitamin B6 supplements provides a concrete example of how cumulative exposure from over-the-counter products can translate into regulatory intervention when consumer understanding of dose and risk is limited [13].

## 5. Conclusions

In this statewide survey of adult CM users in a culturally diverse Australian setting, confidence in CM safety and quality exceeded confidence in its comparative effectiveness, and many participants perceived CMs as lower risk than prescription medicines despite reported adverse effects. Differences by age, ethnocultural background, and CM use intensity suggest that consumer risk perceptions are not uniform. These findings provide a contemporary Victorian baseline to inform targeted clinical communication, consumer education, and future research.

### 5.1. Strengths and Limitations

This study has several strengths, including statewide metropolitan/regional coverage, ethnocultural diversity, and the use of exploratory profiling approaches (LCA and EFA) alongside conventional regression analyses of a contemporary Victorian sample. Several limitations should also be considered. First, the cross-sectional design precludes causal inference. Second, recruitment relied on a non-probability, English-language, venue-based sampling strategy, which may have introduced selection bias and limits the generalisability of the findings. Accordingly, this sample should be understood as a diverse group of Victorian CM users rather than a statistically representative sample of Victorian adults. Third, younger adults were relatively over-represented, which may have influenced the overall estimates of some beliefs. Fourth, all measures, including the CM-related adverse events and their severity, were self-reported with a 12-month recall period and may have been affected by recall and social desirability bias. Fifth, the questionnaire was pilot-tested for clarity but not externally validated against clinical or dispensing data. Finally, residual confounding by unmeasured factors, such as specific diagnoses, health literacy, and health service use, cannot be excluded.

### 5.2. Generalisability

Because this was a non-probability, unweighted sample, the findings are generalisable primarily to English-speaking Victorian CM users recruited from similar community settings and are not estimates of population prevalence. However, the wide metropolitan/regional coverage and ethnocultural diversity mean the relative differences (e.g., by age or use intensity) are likely informative for comparable Australian contexts. Extrapolation to non-English-speaking groups, other regulatory jurisdictions, or clinical subgroups (e.g., multimorbidity/polypharmacy) should be made with caution.

### 5.3. Implications for Practice, Regulation, and Consumer Protection

Strong beliefs about CM safety and efficacy, alongside use for minor ailments but not serious chronic conditions, highlight the need for tailored risk communication within Australia’s regulatory context. Because CM products used in Victoria are regulated through the national TGA/ARTG framework rather than a separate Victorian registration system, opportunities for improvement lie mainly in clearer national labelling, consumer information, and point-of-care counselling. Most CMs are listed rather than registered and are not individually evaluated by the TGA for safety, efficacy, or quality before being made available; instead, sponsors certify compliance and labels are not individually pre-approved, which may contribute to inconsistent interaction/risk warnings and to perceptions that CMs are “lower risk” than prescription medicines [6,21,22]. Four pragmatic actions follow: (1) routine enquiry and counselling by clinicians and pharmacists about CM use, potential interactions, adverse effects, and reporting pathways; (2) targeted messaging for older and frequent users, especially around interactions and high-risk combinations; (3) clearer label warnings or inserts for higher-risk ingredients plus simple icons or QR links to product-specific evidence; and (4) prominent prompts to improve consumer adverse-event reporting and reduce under-reporting [32,40]. The perception profiles identified through the EFA/LCA may also help tailor these messages to groups with different beliefs and information needs.

### 5.4. Future Research

Future research on CMs should prioritise prospective, population-based surveillance of CM exposure and outcomes to quantify the benefits, harms, and interactions over time. Rigorous trials of communication strategies (e.g., pharmacist-led counselling, prompts, shared decision aids) are needed to normalise CM disclosure and improve safety conversations between patients and clinicians. Culturally tailored qualitative studies should map how diverse communities perceive, choose, and integrate CMs, with the findings used to co-design acceptable, effective interventions. Finally, electronic health records and consumer eHealth tools should include structured CM use fields and automated interaction alerts to flag risks at the point of care and support coordinated management.

## Figures and Tables

**Table 1:** Participant characteristics of CM users in Victoria, Australia (N = 447; valid N varies slightly by item).

Characteristic	n	%
Age group (valid n = 432)		
18–24	131	30.3
25–34	87	20.1
35–44	95	22.0
45–54	48	11.1
55–65	33	7.6
65+	38	8.8
Gender (valid n = 447)		
Male	151	33.8
Female	286	64.0
Other/prefer not to say	10	2.2
Ethnicity (valid n = 442)		
Caucasian	168	38.0
Middle Eastern/North African	96	21.7
South Asian	73	16.5
East Asian	32	7.2
Southeast Asian	19	4.3
African	13	2.9
Latin American/Hispanic	8	1.8
Aboriginal/Torres Strait Islander	7	1.6
Māori	1	0.2
Mixed	5	1.1
Prefer not to say	20	4.5
Education (valid n = 446)		
High school or less	120	26.9
Tertiary (undergraduate)	154	34.5
Postgraduate	172	38.6
Residence (valid n = 447)		
Metropolitan	319	71.4
Regional	128	28.6
Overall health status (valid n = 446)		
Excellent/Good	331	74.2
Average	102	22.9
Poor/Terrible	13	2.9
Prescription medicine use (daily) (valid n = 447)		
Yes	193	43.2
No	254	56.8
Presence of Medical Conditions (valid n = 447)		
≥One condition	224	50.1
No conditions	223	49.9

**Table 2:** Patterns of CM use among respondents (N = 447; valid N varies slightly by item).

Variable	n	%
Frequency of use (valid n = 447)		
Daily	278	62.2
Weekly	86	19.2
Monthly	22	4.9
Occasionally (<once/month)	61	13.6
Duration of use (valid n = 447)		
<3 months	69	15.4
3–6 months	72	16.1
7–12 months	60	13.4
>1 year	246	55
Product categories (top 5) (valid n = 447; totals exceed 447 because multiple responses were allowed)		
Vitamin D	237	53
Multivitamin	178	39.8
Magnesium	154	34.5
Iron	151	33.8
Vitamin C	134	30
Recommendation source of use (valid n = 429)		
Healthcare professional	171	39.9%
Self/non-professional	258	60.1%

Product categories are multiple-response items and therefore do not sum to 100%. Recommendation source percentages are based on valid responses only. CMs: Complementary medicines.

**Table 3:** Associations between demographics and perceptions of CMs.

Perception Item	N (Valid)	χ^2^	*df*	*p*-Value	Cramér’s V	q (BH–FDR, Within Predictor Family)
**Gender**						
Medications should have no side effects	425	18.12	6	0.006	0.146	0.1623
**Age groups**						
Effectiveness of CMs	428	27.86	10	0.002	0.18	**0.0085**
CMs more effective than prescription medicines	427	19.84	10	0.031	0.15	0.6393
CMs have lower side effects than prescription medicines	427	34.30	10	<0.001	0.20	**0.0015**
Medications should have no side effects	427	22.75	10	0.012	0.16	0.3483
Patients should be involved in decision-making	426	18.44	10	0.048	0.15	0.3254
**Education level**						
Safety of CMs	436	12.58	4	0.0135	0.12	0.1216
Patients should be involved in decision-making	427	26.502	4	<0.001	0.176	0.6668
**Ethnicity**						
Effectiveness of CMs	435	27.749	6	<0.001	0.194	**0.0036**
Safety of CMs	435	15.869	6	0.018	0.147	0.1094
Medications should have no side effects	428	13.494	6	0.0358	0.136	0.217
Patients should be involved in decision-making	427	30.978	6	<0.001	0.206	**0.0036**
**Health status**						
Effectiveness of CMs	435	23.58	4	<0.001	0.17	**<0.001**
Safety of CMs	435	18.27	4	0.001	0.15	**0.0049**
**Frequency of CM Use**						
CMs have lower side effects than prescription medicines	428	12.03	2	0.002	0.17	**0.0110**

χ^2^ = chi-square; *df* = degrees of freedom; Cramér’s V = effect size. Perceptions were collected on 5-point Likert scales and collapsed to three levels: disagreement (1–2), neutral/unsure (3), agreement (4–5). Gender analyses include male/female only; education = high school or less, undergraduate, postgraduate; ethnicity includes Caucasian/White, Middle Eastern/North African, South Asian, and East Asian (other/prefer not to answer are excluded); residence = metropolitan vs. regional; CM frequency shown = 2 levels (daily/weekly vs. monthly/occasional). N = listwise valid cases. Omnibus p from Pearson’s χ^2^; q (BH–FDR) is computed within each predictor family; for *q* < 0.05, post hoc uses ASR with Holm correction. Sparse expected counts are addressed by prespecified category collapsing. Post hoc details are provided in Supplementary File S3.

**Table 4:** Summary of multivariable associations with CM perceptions.

Characteristics (Reference Category)	Perceived Effectiveness (aOR *, 95% CI, p-Value)	Perceived Safety (aOR *, 95% CI, p-Value)	Perceived Quality (aOR *, 95% CI, p-Value)	Lower Side Effect Risk vs. Same/Higher (aOR *, 95% CI, p-Value)
**Age group (ref: 18–24 years)**				
**25–34 years**	3.04, 1.33–6.97, 0.009	**_**	_	_
**35–44 years**	4.10, 1.78–9.46, <0.001	_	_	_
**45–54 years**	4.29, 1.50–12.31, 0.007	_	_	_
**55–65 years**	4.86, 1.27–18.55, 0.021	_	3.8, 1.39–10.48, 0.01	_
**≥65 years**	_	_	_	0.18, 0.06–0.51, 0.001
**Gender (ref: Female)**				
**Male**	_	_	1.73, 1.09–2.74, 0.021	_
**Education (ref: High school or less)**				
**Postgraduate**	0.32, 0.15–0.67, 0.002	0.33, 0.11–0.97, 0.044	_	_
**Ethnicity (ref: Caucasian/White)**				
**East Asian**	0.28, 0.11–0.72, 0.008	_	_	_
**CM use frequency (ref: Daily/Weekly)**				
**Monthly/Occasional**	0.47, 0.26–0.83, 0.009	_	_	0.36, 0.18–0.72, 0.004

* aOR: adjusted odds ratio. Overall *p*-values are for each predictor within each model; row-level *p*-values are for individual contrasts versus reference category. Notes: Only predictors with *p* < 0.05 are shown; non-significant predictors are omitted for brevity. Measure = OR for ordinal logistic models. Comparative side effect risk (CMs vs. prescription medicine) is modelled as binary outcome due to sparse ‘higher risk’ responses: ‘Lower risk’ vs. ‘About the same’/‘Higher’ (combined). Constants are omitted from summary.

## Data Availability

The study data cannot be publicly shared for reasons of ethics and privacy. Access may be granted upon reasonable request to the corresponding author if deemed appropriate by the investigators. All authors had full access to all study data (including statistical reports and tables).

## Supplementary Materials

The following supporting information can be downloaded at: https://www.mdpi.com/article/10.3390/nu18071077/s1, File S1: Survey Instrument; File S2: STROBE checklist for cross-sectional studies; File S3: Cellwise post-hoc analyses; Figure S1: Demographic profiles by latent class; Table S1: Full list of complementary medicine products reported by participants; Table S2: Associations between usage frequency and perceptions of CMs; Table S3: Multinomial (Higher vs Same; Lower vs Same) estimates; Table S4: LCA item-response probabilities (Disagree/Neutral/Agree) by class for nine perception items.

## Abbreviations

CMs	complementary medicines
TGA	Therapeutic Goods Administration
EFA	exploratory factor analysis
LCA	latent class analysis
GI	gastrointestinal
