# Supplementary material for: Complementary Medicine Use and Perceptions of It in Victoria, Australia: A Statewide Cross-Sectional Survey
Source: Nutrients. 2026 Mar 27;18(7):1077. doi: 10.3390/nu18071077 (PMC13074535; doi:10.3390/nu18071077)
Supplement: Supplementary file 1 [file nutrients-18-01077-s001.zip › nutrients-4200468-supplementary/Supplementary Figure S1.pdf]

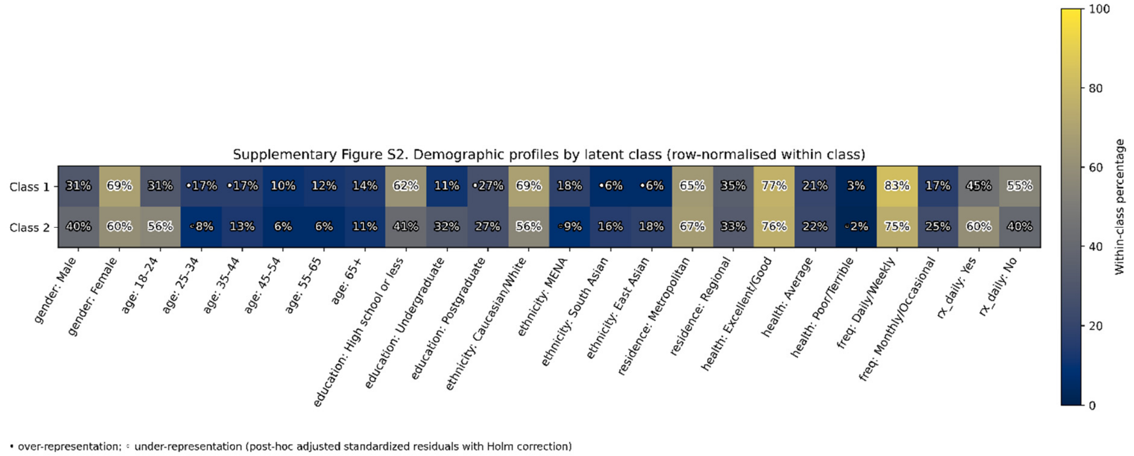

Supplementary Figure S1. Demographic profiles by latent class (row-normalized within-class %). Rows = latent classes; columns = levels within each demographic. Cell values are % of respondents within class; • denotes over-representation, ◦ denotes under-representation (post-hoc adjusted standardized residuals with Holm correction).
