# Supplementary material for: Complementary Medicine Use and Perceptions of It in Victoria, Australia: A Statewide Cross-Sectional Survey
Source: Nutrients. 2026 Mar 27;18(7):1077. doi: 10.3390/nu18071077 (PMC13074535; doi:10.3390/nu18071077)
Supplement: Supplementary file 1 [file nutrients-18-01077-s001.zip › nutrients-4200468-supplementary/Supplementary File S1.pdf]

Use and perceptions of Natural and Complementary Health Products

Thank you for considering this survey, which explores the use and perceptions of natural and complementary health products, including vitamins, minerals, herbal medicines, probiotics, and prebiotics. If you are 18 years or older, currently living in Victoria, Australia, and have used these products in the past 12 months, you are eligible to participate. The survey will take approximately 10–15 minutes to complete, and your responses will remain confidential and anonymous, used solely for research purposes. Participation is voluntary, and you can stop at any time. If you need support, services like Beyond Blue (1300 22 4636) and Lifeline (13 11 14) are available.

1. **Are you currently living in Victoria, Australia?**

   ☐Yes

   ☐No

2. **Are you 18 years old or above?**

   ☐Yes

   ☐No

3. **Are you currently taking, or have you taken any natural or complementary health product during the last 12 months?**

   ☐Yes

   ☐No

If your responses to the above questions were "**Yes**", please continue with the following questions.

4. **How often do you currently use, or have you used natural or complementary health products?**

   ☐Daily

   ☐Weekly

   ☐Monthly

   ☐Occasionally (less than once a month)

5. **For how long have you been using natural or complementary health products?**

   ☐Less than 3 months

   ☐3-6 months

   ☐7-12 months

   ☐More than a year

6. Which of the following natural or complementary health product/s are you currently using or have used in the past? (Select all that apply)

☐Multivitamin

☐Vitamin B

☐Vitamin C

☐Vitamin D

☐Calcium

☐Zinc

☐Iron

☐Magnesium

☐Ginkgo biloba

☐Natural weight loss product

☐Probiotics

☐Glucosamine

☐Echinacea

☐Fish oils

☐Coenzyme Q10

☐St John's Wort

☐Valerian

☐Collagen

☐Other (please specify): _________________

7. Where do you mostly buy natural or complementary health product? (Select one)

☐Supermarket

☐Pharmacy

☐Herbal/Natural product shop

☐Online

☐Other (please specify): _________________

8. Where do you mainly get your information about natural or complementary health products? (Rank in order with number one being the most frequently used)

☐Family/Friends

☐Internet/Media

☐Naturopath/Herbalist

☐Label of the product

☐Pharmacist

☐Medical doctor

☐Other (please specify): _________________

9. How would you prefer to receive information about natural or complementary health products? (Identify up to three most preferred options by ranking them)

☐Online resources

☐Printed materials (brochures, pamphlets)

☐Face-to-face workshops or seminars

☐Consultations with healthcare professionals

☐Mobile application

☐Other (please specify): _________________

10. What are your reasons for using the natural or complementary health product? (Select all that apply)

☐To maintain overall health and well-being

☐To prevent diseases

☐Managing chronic conditions (diabetes, heart disease, migraine, arthritis, etc.)

☐Managing pain

☐To treat a specific disease or symptom

☐Supplementing prescription medication and other conventional treatments

☐To improve physical appearance (e.g., skin, hair)

☐To enhance mental or emotional well-being

☐They work better or just as well as other medicines.

11. Who typically recommends you the natural or complementary health products you use? (Select one)

☐A friend

☐A family member

☐Doctor

☐Pharmacist

☐Naturopath

☐Health food store staff

☐Social media influencer or content creator

☐A sports or gym trainer

☐Other (please specify) ________________

12. How <u>effective</u> do you think the natural or complementary health products you take are?

☐Very effective

☐Moderately effective

☐Slightly effective

☐Not effective

☐Don't know/unsure

13. How <u>safe</u> do you think the natural or complementary health products you take are?

☐Very safe

☐Moderately safe

☐Slightly safe

☐Not safe

☐Don't know/unsure

14. In general, how would you rate the <u>quality</u> of natural or complementary health products?

☐Very high quality

☐High quality

☐Neutral (not sure)

☐Low quality

☐Very low quality

Please choose the option that best matches your perception of natural or complementary products in the following statements.

15. Natural or complementary products are <u>safer</u> than prescription medications.

☐Strongly agree

☐Agree

☐Neither agree nor disagree

☐Disagree

☐Strongly disagree

16. **Natural or complementary products are more effective than prescription medications.**

☐Strongly agree

☐Agree

☐Neither agree nor disagree

☐Disagree

☐Strongly disagree

17. **Medications (both prescribed and over-the-counter) should have no side effects. (Choose the option that best matches your opinion).**

☐Strongly agree

☐Agree

☐Neither agree nor disagree

☐Disagree

☐Strongly disagree

18. **Patients should be involved in decision-making regarding their disease management, including the use of natural or complementary health products. (Choose the option that best matches your opinion).**

☐Strongly agree

☐Agree

☐Neither agree nor disagree

☐Disagree

☐Strongly disagree

19. **For chronic medical conditions (e.g., high blood pressure, diabetes, high cholesterol), I prefer to take natural or complementary health products rather than prescription medication.**

☐Strongly agree

☐Agree

☐Neither agree nor disagree

☐Disagree

☐Strongly disagree

RMIT UNIVERSITY

20. For minor ailments (e.g., cough due to a cold, indigestion, aches and pains), I prefer to take natural or complementary health products rather than a prescription medication.

☐Strongly agree

☐Agree

☐Neither agree nor disagree

☐Disagree

☐Strongly disagree

21. How do you perceive the risk of side effects when using natural or complementary health products compared to prescription medications?

☐Much lower risk (Maybe no risk)

☐Lower risk

☐About the same risk

☐Higher risk

☐Much higher risk

22. Have you experienced any adverse reactions or side effects (e.g., rash, nausea, diarrhea, etc.) to natural or complementary health products?

☐No

☐Yes

☐Unsure

If you answered "Yes" to question 22, please proceed to questions 23–25. If not, kindly continue from question 26.

23. How serious was the reaction?

☐Severe (required hospitalization)

☐Moderate (symptoms required a visit to a doctor or healthcare professional)

☐Mild (no specific treatment required)

☐Please specify the adverse reaction(s) you experienced: ___________

24. What did you do about the reaction?

☐I stopped using the product.

☐I reduced the dose I was taking.

☐I continued taking it as the adverse reaction was mild.

☐I changed to another natural or complementary health products.

☐I sought advice from a healthcare professional.

☐Other (please specify): _________________

25. If you did not report an adverse reaction to a natural or complementary health product, what were the reasons? (Select all that apply)

☐Didn't know where or how to report

☐Believed it was not serious enough

☐Thought it was normal and not worth reporting

☐Felt embarrassed or unsure

☐Other (please specify): _________________

26. How often do you read the labels on natural or complementary health products before using them?

☐Always

☐Often

☐Sometimes

☐Rarely

☐Never

27. Which of the following information on a product label is important to you? (Select all that apply)

☐Ingredients

☐Dosage instructions (how much to take)

☐Warnings (drug interactions)

☐Benefits/claims

☐Expiry date

☐Manufacturer information

☐Other (please specify): _________________

28. How easy do you find it to understand the information provided on the labels of natural or complementary health products?

☐Very easy

☐Easy

☐Neutral

☐Difficult

☐Very difficult

29. Have you ever encountered a warning on a label that stopped you from using one of these products or led you to seek more information before using it? (Select one)

☐Yes, it stopped me from using the product (Please specify the warning if you remember): _________________

☐Yes, it led me to seek more information (Please specify the warning if you remember): _________________

☐No, I don't read labels.

☐No, I read labels but have not been concerned by any warnings.

☐No, I read labels but don't understand them.

☐Other (please specify): _________________

30. How often do you consult a healthcare professional (e.g., doctor, nurse, pharmacist) before using a natural or complementary health product?

☐Always

☐Often

☐Sometimes

☐Rarely

☐Never

31. How satisfied were you with the advice provided by healthcare professionals regarding natural or complementary health products?

☐Very satisfied

☐Satisfied

☐Neutral

☐Dissatisfied

☐Very dissatisfied

32. What improvements would you like to see in the labelling of natural or complementary health products? (Open-ended and optional to answer)

Demographic Information

33. What age group do you belong to?

☐18-24

☐25-34

☐35-44

☐45-54

☐55-64

☐65 or older

34. How do you identify your gender?

☐Male

☐Female

☐Non-binary/Third gender

☐Prefer not to say

☐Prefer to self-describe: _________________

35. What is the highest level of education you have completed?

☐Less than high school

☐High school graduate

☐Some college

☐2 year degree

☐4 year degree

☐post graduate degree

☐Professional degree

☐Doctorate

36. Where is your primary place of residence?

☐Metropolitan

☐Rural/Regional

37. What is your ethnicity?

☐Aboriginal or Torres Strait Islander

☐Australian

☐European

☐East Asian (e.g., Chinese, Japanese, Korean)

☐South Asian (e.g., Indian, Pakistani, Bangladeshi)

☐Southeast Asian (e.g., Vietnamese, Thai, Filipino)

☐Middle Eastern or North African

☐African

☐Pacific Islander (e.g., Fijian, Tongan, Samoan)

☐Māori

☐Latin American or Hispanic

☐Other (please specify): ________________

☐Prefer not to say

38. How would you describe your overall health?

☐Excellent

☐Good

☐Average

☐Poor

☐Terrible

39. Please indicate if you have any of the following medical conditions:

☐Arthritis

☐Asthma

☐Back pain

☐Cardiovascular disease

☐Diabetes

☐Mental health conditions

☐Cancer

☐Chronic obstructive pulmonary disease

☐Liver impairment

☐I don't have any medical conditions.

☐Other (please specify): ________________

40. Do you take any prescription medicines on a daily basis?

☐Yes

☐No

41. If Yes, did you consult your treating practitioner prior to taking the natural or complementary health products?

☐Yes

☐No

You have successfully completed the survey. If you wish to enter the draw to win a $20 voucher and/or participate in a small group discussion (focus group) where you will share your thoughts and experiences about complementary medicines with other participants, please indicate your preferences below and provide your contact details.

☐I would like to enter the draw to win $20 voucher.

☐I would like to participate in the small group discussion.

☐I want to do both.

☐I am not interested in participating further.

If you selected any of the first three options above, please provide your contact details below.

Full Name ___________________________

Email Address ________________________

Phone Number (Optional, for small group discussion invitation) _________________________

End of Survey

Thank you for participating in this survey. Your responses are valuable and will help improve the understanding and regulation of natural and complementary health products.
