# Supplementary material for: Complementary Medicine Use and Perceptions of It in Victoria, Australia: A Statewide Cross-Sectional Survey
Source: Nutrients. 2026 Mar 27;18(7):1077. doi: 10.3390/nu18071077 (PMC13074535; doi:10.3390/nu18071077)
Supplement: Supplementary file 1 [file nutrients-18-01077-s001.zip › nutrients-4200468-supplementary/Supplementary File S2.pdf]

STROBE checklist (cross-sectional studies)

Manuscript: Complementary medicine use and perceptions in Victoria, Australia: a statewide cross-sectional survey

Page references below correspond to the submitted Manuscript Word document.

Item	Checklist item (STROBE)	Manuscript location (page no.)	Notes (if needed)
1a	Indicate the study design in the title.	1	Design stated in title ("statewide cross-sectional survey").
1b	Provide an informative, structured abstract (design, setting, participants, outcomes, results, conclusions).	1	Structured abstract reports design, setting/participants, outcomes, main results and conclusion.
2	Explain scientific background and rationale.	3-5	Background/rationale describe widespread CM use and knowledge/risk gaps.
3	State specific objectives / prespecified hypotheses.	1, 5	Objectives stated (describe CM use/perceptions; examine sociodemographic correlates).
4	Present key elements of study design early in the paper.	1, 5	Study design described early in Methods ("statewide cross-sectional survey").
5	Describe setting, locations, and relevant dates (recruitment, data collection).	1, 5	Setting, recruitment venues and dates stated (Nov 2024–Aug 2025; metro/regional).

6a	Give eligibility criteria and how participants were identified/selected.	5–6	Eligibility, exclusions and recruitment/selection methods described; screening at entry.
6b	Describe sources and methods of participant recruitment/selection.	5–6	Venue-based purposive sampling plus online recruitment described.
7	Clearly define all outcomes, exposures, predictors, potential confounders, and effect modifiers.	5–7	Outcomes are CM perception items; predictors include demographics and CM-use measures.
8	For each variable of interest, give data sources and measurement details.	5–7	Survey instrument described; item formats and pilot testing noted.
9	Describe efforts to address potential sources of bias.	14–15	Non-probability sampling and selection/self-report limitations discussed; unweighted estimates noted.
10	Explain how the study size was arrived at.	8	A priori power/sample size calculation plus achieved sample size.
11	Explain handling of quantitative variables (groupings/cut-points, transformations).	6	Likert collapsing; ethnicity grouping; frequency collapsing; risk item mapping.
12a	Describe all statistical methods (including confounding control).	6–8	Regression models, chi-square tests, multiple-comparison control, etc.
12b	Describe methods for subgroup analyses and interactions (if any).	6	Subgroup comparisons (eg, HCP recommendation; Rx users) described.
12c	Explain how missing data were addressed.	6–7	Available-case approach; imputation sensitivity noted; N varies by item in Fig 1 caption.

12d	If applicable, describe analytical methods accounting for sampling strategy.	8, 15	Non-probability design; unweighted estimates explicitly stated.
12e	Describe any sensitivity analyses.	7, 11	Imputation and sparsity checks described.
13a	Report numbers of individuals at each stage of the study.	6	Because recruitment occurred via open QR/social media links and community venues, the number approached and response rate could not be precisely calculated.
13b	Give reasons for non-participation at each stage.	Not reported	Reasons for non-participation were not quantified because recruitment included open online and venue-based survey access with anonymous participation. Eligibility was enforced via screening questions; records were screened and incomplete entries and potential duplicates were removed prior to finalising the analysis dataset.
13c	Consider use of a flow diagram.	Included in the page 6 of the manuscript	A participant flow diagram is not provided. The final sample included 447 completed surveys; analytic Ns are reported per analysis (tables/figures) due to available-case handling and item-level missingness.

14a	Give characteristics of study participants (demographic/clinical/social) and exposures/confounders.	8, 21	Table 1 provides baseline characteristics.
14b	Indicate number of participants with missing data for each variable of interest.	6–9, 21–24	Item-level valid N noted; consider adding a brief missingness summary table in Supporting Info.
14c	Summarize follow-up time (if relevant).	N/A	Cross-sectional study.
15	Report numbers of outcome events / summary measures.	8–11, 21–24	Perception distributions and associations reported; tables at end.
16a	Give unadjusted and (if applicable) adjusted estimates with precision (eg, 95% CI).	10–11, 24	Adjusted ORs with 95% CIs reported in Table 4.
16b	Report category boundaries when continuous variables were categorized.	6, 21–24	Age groups, frequency groupings, etc.
16c	If relevant, translate relative measures into absolute risk (when meaningful).	8–11, 21–24	Absolute counts/percentages are reported; NNT/NNH not applicable.
17	Report other analyses (subgroups, interactions, sensitivity, ancillary analyses).	11–12	EFA and LCA results; figures at end.
18	Summarize key results with reference to objectives.	11	The first paragraph of the Discussion section
19	Discuss study limitations (potential bias/imprecision; direction/magnitude).	14–15	As the Limitation section
20	Provide a cautious overall interpretation considering objectives, limitations, multiplicity, other evidence.	15–16	The last paragraph of the Discussion section
21	Discuss generalisability (external validity).	15–16	The last paragraph of the Discussion section
22	Give sources of funding and role of funders.	17	This research received no specific grant from any funding agency in the public, commercial or not-for-profit sectors.
