# Supplementary material for: Complementary Medicine Use and Perceptions of It in Victoria, Australia: A Statewide Cross-Sectional Survey
Source: Nutrients. 2026 Mar 27;18(7):1077. doi: 10.3390/nu18071077 (PMC13074535; doi:10.3390/nu18071077)
Supplement: Supplementary file 1 [file nutrients-18-01077-s001.zip › nutrients-4200468-supplementary/Supplementary Tables.pdf]

Supplementary Table S1. Full list of complementary medicine products reported by participants

Product	Number of users	% of users
**Vitamin D**	237	53
**Multivitamin**	178	39.8
**Magnesium**	154	34.5
**Iron**	151	33.8
**Vitamin C**	134	30
**Fish oil/Omega-3**	114	26
**Vitamin B**	99	22.6
**Zinc**	80	18.3
**Calcium**	67	15.3
**Probiotics**	64	14.6
**Collagen**	46	10.5
**Glucosamine**	17	3.9
**Echinacea**	14	3.2
**Ginkgo biloba**	14	3.2
**Coenzyme Q10**	12	2.7
**Turmeric/Curcumin**	6	1.4
**Valerian**	6	1.4
**St John's wort**	5	1.1
**Ashwagandha**	4	0.9
**Folic acid**	3	0.7
**Ayurvedic medicine (herbal mix)**	1	0.2
**Psyllium (fibre)**	1	0.2

Extended list of complementary-medicine (CM) products used in the past 12 months: number of users (n) and column percentage (%). Multiple responses permitted; percentages may exceed 100%. Items ordered by prevalence. Brand names collapsed to generic constituents; category definitions follow the survey instrument (**Supplementary File 1**).

Supplementary Table S2. Associations between usage frequency and perceptions of CMs

Perception Item	Spearman ρ	p-value
**Effectiveness of CMs**	0.20	**< 0.001**
**Safety of CMs**	-0.010	0.840
**Quality of CMs**	0.12	**0.048**
**CMs more effective than prescription medicines**	0.16	**0.002**
**Medications should have no side effects**	0.054	0.300
**Patients should be involved in decision-making**	-0.054	0.274
**Preference for CMs in chronic conditions**	0.042	0.435
**Preference for CMs in minor ailments**	0.012	0.818
**CMs lower side-effect risk than prescription medicines**	0.11	**0.031**

Spearman's rank correlation coefficients (ρ) between usage frequency of complementary medicines (coded as 1=Occasionally, 2=Monthly, 3=Weekly, 4=Daily) and nine perception items. Perceptions were measured on five-point ordinal scales and recoded for analysis. Significant associations ($p < 0.05$) are highlighted. Positive coefficients indicate that more frequent users tended to report stronger agreement or higher ratings on perception items.

Supplementary Table S3. Multinomial (Higher vs Same; Lower vs Same) estimates.

Outcome	Predictor	Contrast vs reference	Measure	Estimate (95% CI)	p-value
**Perceived risk (Higher risk vs same)**	Age group	65+ vs 18–24	RRR	5.01 (1.90–13.18)	0.001
**Perceived risk (Higher risk vs same)**	CM use frequency	Monthly/Occasional vs Daily/Weekly	RRR	2.78 (1.46–5.29)	0.002

Notes. Only predictors with $p < 0.05$ are shown; non-significant predictors are omitted for brevity. Reference categories: Age 18–24; CM use frequency Daily/Weekly; Daily prescription medicine use No. Measure = RRR for multinomial models. Risk outcome modelled as binary (Lower vs About the same) due to sparse "Higher" counts; estimates adjusted for age group, gender, education, ethnicity, residence, self-rated health, CM-use frequency (2-level), and daily prescription use. Constants are omitted from the summary.

Supplementary Table S4. LCA item-response probabilities (Disagree/Neutral/Agree) by class for nine perception items (complete cases, N = 178; selected K = 2; entropy = 0.791)

Item	Class 1: Disagree/Low	Class 1: Neutral/Unsure	Class 1: Agree/High	Class 2: Disagree/Low	Class 2: Neutral/Unsure	Class 2: Agree/High
**CMs more effective than prescription medicines**	0.122	0.552	**0.326**	0.636	0.363	**0.001**
**CMs risk vs prescription medicines (lower↔higher)**	0.009	0.167	**0.824**	0.046	0.187	**0.767**
**Effectiveness of CMs**	0.088	0.017	**0.895**	0.202	0.110	**0.689**
**Medications should have no side effects**	0.100	0.217	**0.683**	0.505	0.239	**0.256**
**Patients involved in decision-making**	0.077	0.384	**0.539**	0.687	0.162	**0.153**
**Prefer CMs in chronic conditions**	0.000	0.146	**0.854**	0.154	0.069	**0.776**
**Prefer CMs in minor ailments**	0.171	0.432	**0.397**	0.830	0.170	**0.000**
**Quality of CMs**	0.000	0.000	**1.000**	0.047	0.016	**0.938**
**Safety of CMs**	0.000	0.000	**1.000**	0.031	0.031	**0.938**

Values are within-class probabilities for each response category; columns in bold highlight Agree/High. Items were collapsed to three ordered categories (Disagree/Low = 1–2; Neutral/Unsure = 3; Agree/High = 4–5). The comparative risk item was mapped to Higher / About the same / Lower and similarly collapsed. LCA estimated with EM (multiple random starts; local independence). Selected solution by BIC with the guardrail that no class <10% of N. Class proportions: 63.7% (Class 1) and 36.3% (Class 2). Entropy = 0.791.
